# News Items of General Interest

**Published:** 1914-10

**Authors:** 


### News Items of General Interest.

A new county hospital which will cost $24,469 is being erected at El Paso.

The Guyton-Nichols Sanitarium of Plainview, Texas, is to have an addition of twenty rooms in the near future.

Dr. H. A. Barr of Beaumont was one of the Texas physicians who attended the Congress of Surgeons in London.

Dr. Sherrill of Caldwell is having his residence remodeled and enlarged into a sanitarium for the benefit of his patients.

We announce with deep regret the death of Mr. Jesse Battle, of Battle & Co., St. Louis. Mr. Battle's death occurred on the 16th of September.

Dr. E. M. Outlaw of Palestine has been appointed to succeed Dr. O. H. Judkins as field director of the Hookworm Commission. Dr. Judkins has charge of the Texas health train.

Dr. N. T. Moore of El Paso is being held as a prisoner in Culician, Sinaloa, Mexico, because he performed an operation upon a resident of that city which was followed by his death.

Drs. Petty and Wallace of Memphis, Tenn., are making valuable and extensive additions to their sanitarium. When complete this will be one of the most up-to-date sanitariums in the country for the treatment of drug addicts.

Sanger Brothers of Waco have employed Dr. J. R. Ferrell to deliver a series of addresses to their employes on the subjects of health and hygiene. This is a good plan; let us hope that other large firms will do likewise.

Dr. J. W. Torbett of Marlin scored another honor for Texas and Texas physicians when he was elected to the presidency of the American Electro Therapeutical Association on September 19. He succeeds Dr. George Phaler of Philadelphia.

A two days' session of the Texas Eclectic Medical Association will be held in Dallas, commencing October 28. The Texas Homeopathic Medical Association will be in session at this time and a joint banquet will be given on the opening night.

Drs. W. H. Neely, T. M. Jarmon, and W. F. Alexander are justly proud of the new Terrell Sanitarium, which is said to be "the last word in comfort and convenience," and notwithstanding it is as yet incomplete as regards the matter of interior decoration, the rooms are already full.

The Floeckinger Sanitarium will in the future be known as the City Hospital of Taylor. Dr. F. C. Floeckinger, Dr. E. F. Mikeska, and Dr. Edmond Doak are the physicians in charge. This hospital is a marvel of completeness, and with such an able staff we predict wonderful success for it.

Doctors desiring a change of climate for their tubercular patients would do well to write the Albuquerque Sanatorium of New Mexico for their descriptive pamphlet, which gives all information necessary. This pamphlet is handsomely gotten up and gives one a splendid idea of the surrounding country and the completeness of the institution.

The San Angelo Sanatorium is calling the attention of the doctors of the State to the fact that their institution has, by real merit, placed itself in the front ranks of tubercular sanatoria. A descriptive circular is in process of preparation and those who desire to know more about this health resort should write to Dr. T. K. Proctor for this folder.

Dr. W. R. Newton is being congratulated upon the splendid structure that he has recently erected in Cameron. This new hospital is said by those who have seen it to be one of the most modern and best equipped in the South. Surely, Cameron is proud of a $75,000 hospital, for few places so small can boast of such a large and well equipped sanitarium.

Drs. William M. Morgan, Edgar Smith, T. P. Coopwood, A. A. Ross, and W. H. Obanion are responsible for the new fireproof hospital that is being erected in Lockhart. The building is to be complete in every respect and an elevator, septic tanks, water and electric lighting systems will make it one of the most modern structures in that part of the country.

Many physicians will be interested in knowing that the Bremerman Sanatorium (Inc.) has been organized for the purpose of erecting at Potash Sulphur Springs, Lawrence, Ark., the only institution in America devoted exclusively to urological surgery. The consulting staff includes some of the most prominent doctors in the United States. Dr. Lewis Wine Bremerman of Chicago will be surgeon-in-chief.

Jim Kelly of the *Chicago Herald* conceived the plan of a Christmas ship to Europe. A. P. Goodman of the *Houston Post* has offered to supply Texas grown pecans and peanuts as Texas' part of the cargo of the Christmas ship. And he has pledged 1,000,000 quarter pounds of these delicious and nutritious dainties for the little people on the other side of the ocean. Let each of us give him our aid in this matter.

The negroes of San Antonio propose to build a hospital for themselves; the purpose of the hospital is to educate the negro women that they in turn may go out and nurse and teach their own race the value of clean and healthful surroundings. This is a splendid idea, and we do not doubt that the white physicians of San Antonio will render them all the assistance and encouragement necessary to make their dreams a reality.

Dr. W. B. Bizzell has been appointed President of the A. and M. College, and the *Houston Post* has the following complimentary notice: "Dr. Bizzell is peculiarly adapted for the presidency. He is a native of Texas, educated at Baylor University with post-graduate degrees from Chicago and Columbia universities. He is in early middle age, earnest in purpose and vigorous in action. His life has been spent in educational work and he thoroughly knows the game. His success at the College of Industrial Arts at Denton proves his ability and frees the future at the Agricultural and Mechanical College from any uncertainty. Incidentally, his election will promote the cordial relations which have always existed between the State's great and growing industrial institutions—the college for young women at Denton and the one for men at College Station. We feel gratified that Dr. Bizzell has accepted the position and look with confidence to his successful administration."

---

Dr. James A. Hill, president of the Harris County Medical Society, is home after a month's absence in the North and East.

